# Contrasting Perspectives of Anesthesiologists and Gastroenterologists on the Optimal Time Interval between Bowel Preparation and Endoscopic Sedation

**DOI:** 10.1155/2015/497176

**Published:** 2015-06-17

**Authors:** Deepak Agrawal, Javier Marull, Chenlu Tian, Don C. Rockey

**Affiliations:** ^1^Division of Digestive and Liver Diseases, University of Texas Southwestern Medical Center, Dallas, TX 75390, USA; ^2^Department of Anesthesiology, University of Texas Southwestern Medical Center, Dallas, TX 75390, USA; ^3^Department of Internal Medicine, Medical University of South Carolina, Charleston, SC 29425, USA

## Abstract

*Background*. The optimal time interval between the last ingestion of bowel prep and sedation for colonoscopy remains controversial, despite guidelines that sedation can be administered 2 hours after consumption of clear liquids.* Objective*. To determine current practice patterns among anesthesiologists and gastroenterologists regarding the optimal time interval for sedation after last ingestion of bowel prep and to understand the rationale underlying their beliefs.* Design*. Questionnaire survey of anesthesiologists and gastroenterologists in the USA. The questions were focused on the preferred time interval of endoscopy after a polyethylene glycol based preparation in routine cases and select conditions.* Results*. Responses were received from 109 anesthesiologists and 112 gastroenterologists. 96% of anesthesiologists recommended waiting longer than 2 hours until sedation, in contrast to only 26% of gastroenterologists. The main reason for waiting >2 hours was that PEG was not considered a clear liquid. Most anesthesiologists, but not gastroenterologists, waited longer in patients with history of diabetes or reflux.* Conclusions*. Anesthesiologists and gastroenterologists do not agree on the optimal interval for sedation after last drink of bowel prep. Most anesthesiologists prefer to wait longer than the recommended 2 hours for clear liquids. The data suggest a need for clearer guidelines on this issue.

## 1. Introduction

Polyethylene glycol electrolyte solution (PEG, Golytely) is widely used to cleanse the bowel before colonoscopy. Recent studies advocate use of “split dosing” (taking a portion the night before and finishing the remainder in the morning several hours prior to scheduled colonoscopy) because it leads to superior bowel preparation, higher polyp detection, and greater patient compliance [1–3]. A shorter interval of 3–5 hours between the time of ingestion of the last PEG solution and start of colonoscopy also predicts optimal bowel preparation quality [4, 5]. Despite these data, split dosing is currently not widely implemented. The primary reason for this appears to be that, because of concerns over potential pulmonary aspiration from residual gastric contents, many physicians recommend waiting at least 6–8 hours after finishing drinking colon prep before sedation for the procedure [3, 6]. Unfortunately, this practice is difficult from a practical standpoint. For a patient scheduled to have a colonoscopy at 9 AM, they would have to be up at 3–5 AM to drink their bowel prep.

Current recommendations concerning the most appropriate fasting interval appear to come primarily from anesthesiologists. American Society of Anesthesiologists (ASA) guidelines that state that* healthy* patients may take clear liquids up to 2 hours before administration of anesthesia [7]. It has been our experience that most gastroenterologists consider PEG based solutions to be a clear liquid. This would suggest that based on ASA guidelines a 2-hour time interval between ingestion of PEG and sedation is appropriate. Potential divergence of opinion among gastroenterologists and anesthesiologists has often been inferred but not quantified or openly discussed. We conducted a survey study to understand practices and beliefs of anesthesiologists and gastroenterologists regarding the optimal time interval between ingestion of the last bowel preparation and performance of endoscopy.

## 2. Methods

A questionnaire was constructed by interviewing gastroenterologists and anesthesiologists from our institution and having them answer questions in an open-ended manner. Responses were used to construct the final questionnaire, which was then distributed to 30 gastroenterologists and anesthesiologists and their directors from 3 different institutions; subsequently, feedback from this group was used to perform linguistic, internal, and external validation. The questionnaire was then distributed to chiefs and/or directors of anesthesiology and gastroenterology at academic hospitals in the United States by sending them direct emails. We chose to focus the survey on chiefs and directors since they are considered experts in their fields and dictate policies at their respective institutions. The academic hospitals we contacted were the gastroenterology fellowship programs listed by the American College of Gastroenterology.

The questions in the survey were as follows. (1) How many hours do you wait to perform moderate or deep sedation after a patient has finished drinking their PEG based bowel prep? (2) If you wait >2 hours, why?: I do not consider PEG solution (Golytely) as a clear liquid; I am concerned about the volume of liquid ingested; the risk of aspiration is low but it causes more lung injury if aspirated; there is no data on safety so I take a more conservative approach. (3) If the patient has a history of diabetes (without gastroparesis), proven gastroparesis, or gastroesophageal reflux disease (GERD), would the time interval change; that is, would you wait longer?

Data were summarized using descriptive statistics. Response rates were calculated as number of questionnaires with responses divided by number of questionnaires distributed. Comparisons between groups were measured with the *Z* test of proportions.

## 3. Results

The survey was distributed to 130 gastroenterologists and responses were received from 112, for a response rate of 86%. For the anesthesiologists, the questionnaire was sent to 120 anesthesiologists and responses were received from 109, for a response rate of 91%. At the centers participating in the study, the method of sedation administered was at the discretion of the endoscopist. Deep sedation was administered by anesthesiology provider and moderate sedation by the nurses under supervision of the endoscopist.

Among gastroenterologists, 83 out of 112 (74%) deemed 2 hours a sufficient interval between last ingestion of PEG and the start of colonoscopy compared to only 4 out of 109 (4%) anesthesiologists (Table 1). More anesthesiologists considered 4 hours (66%) and 6 hours (24%) to be the optimal time interval (Table 1) (*P* < 0.05 for the difference between gastroenterologists and anesthesiologists who would wait 2 hours after finishing a colonoscopy prep). The most common reason for choosing a time interval >2 hours was that PEG was not considered a clear liquid (81%) (Table 2).

**Figure 1:**
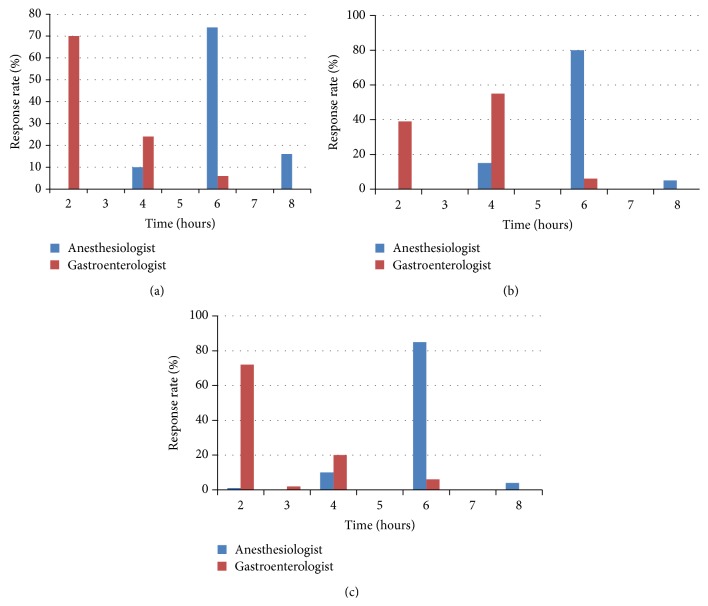
(a) Optimal time interval between last ingestion of bowel preparation and starting endoscopy in* diabetics.* (b) Optimal time interval between last ingestion of bowel preparation and starting endoscopy in patients with* gastroparesis*. (c) Optimal time interval between last ingestion of bowel preparation and starting endoscopy in patients with* reflux.*

For patients with history of diabetes (without gastroparesis), 72% of anesthesiologists preferred to wait longer, compared to only 2% of gastroenterologists (*P* < 0.05). For a patient with history of GERD, 60% of anesthesiologists preferred to wait longer, while no gastroenterologist believed a longer duration of waiting was required (*P* < 0.05). For a patient with history of gastroparesis, 58% of anesthesiologists preferred to wait longer, while 33% of gastroenterologists preferred to wait longer (*P* < 0.05). The proportions of physicians choosing to wait specific time intervals varied among the three clinical scenarios (Figures 1(a), 1(b), and 1(c)).

## 4. Discussion

The increasing use of deep sedation for colonoscopies has greatly increased collaboration between gastroenterologists and anesthesiologists. It is therefore essential that the two groups of providers agree on what constitutes appropriate management of patients undergoing colonoscopy. Our survey found a very significant disconnect between anesthesiologists and gastroenterologists regarding appropriate time interval for administration of PEG and performance of sedation. Specifically, anesthesiologists preferred to wait longer intervals (4–6 hours) after ingestion of bowel preparation prior to sedation compared to gastroenterologists (2 hours).

Our results have important practical implications. Despite strong evidence that split bowel preparations result in better colon preparation and hence increased adenoma detection rate [8], practitioners remain hesitant to prescribe it, apparently because most anesthesiologists recommend waiting 4–6 hours for sedation after ingestion of bowel preparation. This increased waiting results in conflicts in endoscopy scheduling, affects flow in the endoscopy lab, and may impede patient care [9]. For example, many endoscopy centers use split-prep only for patients scheduled in the afternoon to avoid having patients get up at 2 AM or 4 AM to finish the second dose.

Our study also raises a fundamental question: is PEG bowel prep (Golytely) a clear liquid? By definition, polyethylene glycol powder mixed in water is a clear liquid since the solution is transparent, but most anesthesiologists and some gastroenterologists who preferred to wait longer than 2 hours after did not consider it a clear liquid. One explanation is that even though PEG “looks” clear, it does not “behave” as a clear liquid. PEG solution is hyperosmotic and concern is that aspiration of PEG solution may be more dangerous than aspiration of gastric contents. There are reported cases of pulmonary edema and chemical pneumonitis after aspiration of PEG solution [10–13]. However, most of these cases were associated with administration of PEG via NG tube and aspiration occurred even before sedation. Interestingly, in our study, none of the anesthesiologists expressed that concern.

Another concern has been that the large volume of liquid ingested may increase aspiration risk. However, the actual gastric residual volume two hours after ingestion of bowel prep and in overnight fasting patients has been shown to be similar [14–16]. In a recent prospective study of 49 patients, gastric residual volume was found to be less than 20 mL, 2-3 hours after drinking a PEG prep [17]. The ASA also states that the “volume of ingested liquid is less important than the type of liquid ingested.”

A further finding of our study was that for conditions which can possibly delay gastric emptying such as diabetes and gastroparesis an overwhelming majority of anesthesiologists favor the conservative approach of lengthening wait times to 6 hours or even 8 hours whereas the majority of gastroenterologists remain at 2 hours unless there is known gastroparesis. Although data is limited, recent studies do not substantiate longer wait intervals in diabetics without gastroparesis. A population-based cohort study showed that gastroparesis occurs in only 1% of type 2 diabetics and 5.2% of type 1 diabetics over a 10-year time period [18]. Interestingly, even with an established diagnosis of gastroparesis, 50% liquid emptying time has been reported to be much shorter than 2 hours [19, 20].

Waiting a longer period of time between PEG and procedure for a patient who has gastroesophageal reflux disease is controversial. Symptoms of reflux disease are exceedingly common so this issue is especially pertinent. Remarkably, 65% of anesthesiologists but no gastroenterologist in the survey considered history of reflux to be important. It should be emphasized that there are no data available to support the premise that gastroesophageal reflux disease increases the risk of gastric aspiration.

Another issue that may influence anesthesiologists' decision to wait longer is the perceived responsibility and medicolegal liability if pulmonary aspiration occurs. Aspiration is considered a complication of sedation and if the anesthesiologist believes that waiting longer than 2 hours decreases this risk, then they are likely to take a more cautious route. A gastroenterologist, on the other hand, is primarily concerned about quality of the colonoscopy and detection of polyps and so favors a more aggressive preparation and waiting a shorter time interval before colonoscopy. There is a misalignment of risks and rewards for gastroenterologists and anesthesiologists, and they predictably respond to it.

Our study has limitations. First, our sample size is small and limited to the opinions of the chiefs and directors of gastroenterology and anesthesiology at academic institutions. However, we surmise that our respondents are also more likely in a position to guide policies and recommendations. Furthermore, the institutions we contacted are training programs for future gastroenterologists and anesthesiologists, so the opinions of key leaders at these institutions would likely be passed on as teachings and the practices would continue. Second, colonoscopies performed at these academic institutions represent only a small fraction of colonoscopies performed in the USA, so the opinions of the respondents do not necessarily reflect the practice patterns in the community. Third, we did not assess the knowledge of the anesthesiologists regarding importance of a shorter time interval between ingestion of bowel preparation solution and performance of colonoscopy. Thus, it is possible that well-informed anesthesiologists might be more likely to allow a shorter time interval. Notwithstanding, this would not change the data captured and may suggest a need for further education about bowel preparation.

In this study, we have shown how two members of the same team, anesthesiologists and gastroenterologists, would manage the same patient differently, guided by their habits, experiences, and interests. This observation had often been stated but rarely acknowledged or quantified, thus creating a barrier to a meaningful dialogue between them. We hope that our findings will provide the impetus for the two societies to come together and develop joint guidelines on these important issues.

## 5. Conclusions

Anesthesiologists and gastroenterologists do not agree on the interval for sedation after last drink of bowel prep. Most anesthesiologists prefer to wait longer than the recommended 2 hours for clear liquids. There is an urgent need for clearer guidelines on this issue for optimal management of patients undergoing colonoscopy.

## Figures and Tables

**Table 1:** Optimal time interval between bowel preparation and sedation.

	Time interval between finishing bowel prep and sedation (hrs)							Total
	2	3	4	5	6	7	8	
Anesthesiologists	4 (4%)		72 (66%)		26 (24%)		7 (6%)	109 (100%)
Gastroenterologists	83 (74%)	2 (2%)	20 (18%)		7 (6%)			112 (100%)

**Table 2:** Reasons given by respondents for waiting >2 hours.

	Anesthesiologists *N* = 105/109 (96%)	Gastroenterologists *N* = 29/112 (26%)
PEG is not a clear liquid	81%	52%
Concern about volume ingested	32%	5%
PEG causes more lung injury	0	3%
No data on safety	5%	38%
